# NEIL3 may act as a potential prognostic biomarker for lung adenocarcinoma

**DOI:** 10.1186/s12935-021-01938-4

**Published:** 2021-04-20

**Authors:** Cui Zhao, Jian Liu, Haomiao Zhou, Xin Qian, Hui Sun, Xuewen Chen, Miaosen Zheng, Tingting Bian, Lei Liu, Yifei Liu, Jianguo Zhang

**Affiliations:** 1 Nantong University, Nantong, 226001 China; 2 Department of Pathology, Affiliated Hospital of Nantong University, Nantong, 226001 China; 3 Department of Chemotherapy, Affiliated Hospital of Nantong University, Nantong, 226001 China; 4 Department of Orthopedics, Second People’s Hospital of Jingmen, Jingmen, 448000 China

**Keywords:** Bioinformatics, Lung adenocarcinoma, NEIL3, Real-time quantitative PCR, Immunohistochemistry, Prognostic signature

## Abstract

**Background:**

Lung adenocarcinoma (LUAD) is the leading cause of cancer-related death. This study aimed to develop and validate reliable prognostic biomarkers and signature.

**Methods:**

Differentially expressed genes were identified based on three Gene Expression Omnibus (GEO) datasets. Based on 1052 samples’ data from our cohort, GEO and The Cancer Genome Atlas, we explored the relationship of clinicopathological features and NEIL3 expression to determine clinical effect of NEIL3 in LUAD. Western blotting (22 pairs of tumor and normal tissues), Real-time quantitative PCR (19 pairs of tumor and normal tissues), and immunohistochemical analyses (406-tumor tissues subjected to microarray) were conducted. TIMER and ImmuCellAI analyzed relationship between NEIL3 expression and the abundance of tumor-infiltrating immune cells in LUAD. The co-expressed-gene prognostic signature was established based on the Cox regression analysis.

**Results:**

This study identified 502 common differentially expressed genes and confirmed that NEIL3 was significantly overexpressed in LUAD samples (P < 0.001). Increased NEIL3 expression was related to advanced stage, larger tumor size and poor overall survival (*p* < 0.001) in three LUAD cohorts. The proportions of natural T regulatory cells and induced T regulatory cells increased in the high NEIL3 group, whereas those of B cells, Th17 cells and dendritic cells decreased. Gene set enrichment analysis indicated that NEIL3 may activate cell cycle progression and P53 signaling pathway, leading to poor outcomes. We identified nine prognosis-associated hub genes among 370 genes co-expressed with NEIL3. A 10-gene prognostic signature including NEIL3 and nine key co-expressed genes was constructed. Higher risk-score was correlated with more advanced stage, larger tumor size and worse outcome (*p* < 0.05). Finally, the signature was verified in test cohort (GSE50081) with superior diagnostic accuracy.

**Conclusions:**

This study suggested that NEIL3 has the potential to be an immune-related therapeutic target and an independent predictor of LUAD prognosis. We also developed a prognostic signature for LUAD with a precise diagnostic accuracy.

**Supplementary Information:**

The online version contains supplementary material available at 10.1186/s12935-021-01938-4.

## Background

Lung cancer, accounting for almost one-quarter of all cancer deaths, has become more common than breast, brain, colorectal, and prostate cancers combined and is now the leading cause of cancer-related death globally [1]. Lung adenocarcinoma (LUAD) is the most frequent pathological subtype, accounting for nearly 45% of lung cancer cases [2]. The 5-year relative survival rate of LUAD is only 5% because about 57% of patients are diagnosed with advanced stage and metastatic disease [3, 4]. Patients with LUAD have improved overall survival (OS) after surgery and radiotherapy when diagnosed sooner. With the development of immune checkpoint inhibitors (atezolizumab, pembrolizumab, nivolumab, etc.), the survival of LUAD patients has significantly improved; thus, such treatments have attracted considerable attention [5, 6]. However, limited data are available regarding the relationship between biomarkers and immune responses. Therefore, exploring and identifying effective immune-related biomarkers for LUAD to reduce the mortality rates and develop innovative targeted therapies remains crucial.

In the past ten years, researchers have conducted new studies and explored meaningful genes using bioinformatics techniques. Here we identified 502 commonly expressed genes from three LUAD datasets (GSE32863, GSE33532, and GSE43458). After an extensive literature review, we found that DNA endonuclease VIII-like 3 (NEIL3) is a very promising DNA glycosylase [7]. DNA glycosylase is one kind of enzymes to initiate the base excision repair (BER) pathway, recognize, and excise damaged bases from DNA [7–9]. NEIL3 has a glycolysis domain and apurinic/apyrimidinic lyase activity that excises damaged bases and generates a single-strand break [7, 9]. NEIL3 repairs telomere oxidative damage and protects telomere integrity during the S phase to enable accurate chromosome segregation in actively dividing cells [10]. Some findings based on a Neil3−/− mice model indicated that the NEIL3 mutation was linked to impaired B cell function and severe autoimmunity [11]. NEIL3 promotes the proliferation of cardiac fibroblasts and neural progenitor cells in the brain and tends to be overexpressed in cells with a high proliferative capacity, such as cancer cells and those in the bone marrow [8, 12–14]. The transcription of NEIL3 as a cell cycle dependent gene is regulated by BRG1 in breast cancer cells [15, 16]. Moreover, NEIL3 is highly expressed in primary malignant melanomas, which may induce metastatic tumors [17]. Much evidence suggests that NEIL3 as a DNA repair gene is connected to tumorigenesis, therapy resistance, and poor prognosis in astrocytoma [18, 19]. Evidence has shown that NEIL3 participates in regulating the cell proliferation process. However, the association between NEIL3, cancer prognosis, and immune response in LUAD is unclear.

Here we analyzed the correlation between NEIL3 expression and the clinical characteristics, and the prognosis of LUAD patients based on the gene expression and clinicopathology data of three different cohorts [The Cancer Genome Atlas (TCGA), Gene Expression Omnibus (GEO), and our patient group]. We also investigated the relationship between NEIL3 expression and tumor-infiltrating immune cells using Immune Cell Abundance Identifier (ImmuCellAI) and Tumor Immune Estimation Resource (TIMER). To better understand the role of NEIL3 in LUAD development, we performed a gene set enrichment analysis (GSEA) and screened out 370 NEIL3 co-expressed genes. Importantly, nine hub genes were independent predictors for LUAD; thus, we attempted to construct a prognostic signature and validate its prognostic accuracy in other cohorts.

## Materials and methods

### Human tissue samples

Twenty-two pairs of frozen LUAD and adjacent noncancerous tissues obtained from the Affiliated Hospital of Nantong University between March 2018 and June 2019 were subjected to real-time quantitative polymerase chain reaction (RT-qPCR) and western blotting. Fresh tissues were frozen in liquid nitrogen and stored at − 80 °C until the experiment. LUAD tissue microarray and clinical data for 406 tumor samples and 50 normal lung tissue samples were derived from the Pathology Department of the Affiliated Hospital of Nantong University and used as our cohort. The inclusion criteria were as follows: (1) lobectomy and pneumonectomy with mediastinal lymph node dissection or sampling and (2) tumor diagnosed as invasive lung adenocarcinoma by postoperative pathology examination. The exclusion criteria were as follows: (1) no mediastinal lymph node dissection or sampling; (2) a history of other malignancies; (3) miss key clinical information such as age, sex, TNM stage, overall survival, distant and lymph node metastasis. Informed consent was obtained from all patients before the study. The ethical committee of Affiliated Hospital of Nantong University approved the study (number: 2018-L068), which was also conducted according to the Declaration of Helsinki.

### Data resources and preprocessing

The GEO cohort included five gene expression datasets (http://www.ncbi.nlm.nih.gov/geo). GSE33532, GSE30219, and GSE43458 were used to screen out differentially expressed genes (DEGs) between LUAD and adjacent lung tissues by GEO2R (logFC > 1; adjusted *p* < 0.001), which included 40 LUAD tissues and 20 adjacent lung tissues, 85 LUAD tissues and 14 adjacent lung tissues and 80 LUAD tissues and 30 adjacent lung tissues, respectively. The GEO cohort also included GSE31210 (226 LUAD tissues and 20 adjacent lung tissues) and GSE50081 (127 LUAD tissues of stage I and II), which were used to explore the relationship between NEIL3 expression and the clinical outcomes of LUAD patients.

We utilized the gene expression profile and clinical information of the TCGA cohort (workflow type: HTSeq-FPKM; https://portal.gdc.cancer.gov/projects), and determined NEIL3 gene expression using R software (version: 3.5.3) and Strawberry Perl (version: 5.30.2.1). The TCGA cohort included 293 tumor samples and 54 normal lung tissues after the elimination of samples for which key clinical information was missing such as age, sex, tumor-node-metastasis stage (according to the 8th edition of the AJCC TNM staging system), overall survival, distant and lymph node metastasis. Meanwhile cases with a follow-up time of less than 90 days were deleted. Our work was performed in accordance with the TCGA publication requirements.

### Functional enrichment analyses

GSEA, a powerful calculation software (http://software.broadinstitute.org/gsea/index.jsp) based on Gene Ontology (GO) and the Kyoto Gene and Genomic Encyclopedia (KEGG), was used to investigate the possible biological functions of NEIL3. The cohort of LUAD patients was divided into high and low NEIL3 expression groups by median values. The gene sets used in this work (c2.cp.kegg.v5.2.symbols.gmt) were downloaded from the Molecular Signatures Database (http://software.broadinstitute.org/gsea/msigdb/index.jsp) [20]. We performed functional enrichment of the NEIL3 and co-expressed genes using the Bohao Online Enrichment Tool (http://enrich.shbio.com/) [21]. When a false discovery rate (FDR) and the nominal *p* were less than 0.05, the enrichment results were deemed statistically significant.

### Immune infiltrates analysis

TIMER is a user-friendly web tool that is used to investigate the molecular characterization of tumor immune system interactions including six major analytic modules (https://cistrome.shinyapps.io/timer/). We evaluated the correlation between NEIL3 expression and the abundance of the six tumor-infiltrating immune subsets in LUAD: B cells, CD4+ T cells, neutrophils, CD8+ T cells, dendritic cells (DCs), and macrophages [22]. ImmuCellAI has a powerful ability for tumor-immune infiltration estimation, especially in the abundance of 18T-cell subsets (http://bioinfo.life.hust.edu.cn/ImmuCellAI#!/) [23]. A gene set signature of TCGA LUAD patients was uploaded to the website. ImmuCellAI predicted the abundance of 24 immune cells in the sample including 18T-cell subsets. We measured the immune response of 24 immune cells to evaluate their association with NEIL3 expression in LUAD and performed “vioplot” package to visualize the data. At values of *p* < 0.05, the results were considered statistically significant.

### Predictive signature construction and risk score calculation

The Cox proportional hazards model is often applied to survival analyses. We selected out nine prognostic signatures from among the 10 hub genes via “Survival” package and Cox regression analysis. We also performed a multivariate Cox regression analysis including NEIL3 and nine prognostic signatures, and constructed a 10-gene prognostic model to evaluate individual survival risk as follows: risk score = 0.00043 × *NEIL3* expression level + (− 0.00907) × *CCNB2* expression level + 0.00357 × *CDK1* expression level + (− 0.04486) × *CDC45* expression level + 0.05000 × *BUB1B* expression level + (− 0.04195) × *BUB1* expression level + (− 0.0003) × *KIF23* expression level + 0.047506 × *CCNA2* expression level + (− 0.00037) × *UBE2C* expression level + 0.1721 × *NCAPG* expression level. The optimal cutoffs and related specificity and sensitivity from receiver operating characteristic (ROC) curves were determined using a conventional method. Values of *p* < 0.05 were considered statistically significant.

### Quantitative real time polymerase chain reaction

All reactions were performed on a Mastercycler ep realplex (Eppendorf, Hamburg, Germany). The reaction conditions were 95 °C for 5 s, 60 °C for 30 s, and 72 °C for 1 min for a total of 40 cycles. GAPDH was used as the internal control. The RT-qPCR primer sequences were as follows: NEIL3 forward primer, 5′-TACAGGTGCCGTAAAGCAGG-3′ and reverse primer, 5′-GCGAGGGCTGTCAGGATTTA-3′; GAPDH forward primer, 5′-GATCATCAGCAATGCCTCCTG-3′; and reverse primer, 5′-GAGTCCTTCCACGATACCAAAG-3′.

### Western blotting assay

Fresh tissues and protein lysis buffer (including protease inhibitors) were added in homogenizers and grinded fully for extracting proteins. Protein was measured using the bicinchoninic acid method, and separated on a 12% sodium dodecyl sulfate polyacrylamide gel via electrophoresis (cat. #XP00100BOX; Thermo, USA). The protein was then transferred onto polyvinyl difluoride membranes (cat. #88518; Thermo), and the membranes were blocked with 5% skim milk in Tris-buffered saline with Tween-20. After the membranes were incubated with rabbit anti-NEIL3 (1:2000 dilution; cat. #PA5-51022; Thermo) or β-actin overnight at 4 °C (1:10000 dilution; cat. #AM4302; Thermo) and then incubated with anti-rabbit secondary antibodies (1:10,000 dilution; cat. #A32733; Thermo) or anti-mouse secondary antibodies (1:2000 dilution; cat. #A-10654; Thermo) for 1 h. The enhanced chemiluminescence technique was used to develop the signals. The density of the protein bands was quantified by ImageJ software (National Institutes of Health, Bethesda, MD) and normalized to β-actin. Relative protein levels were calculated as the density ratios of interest protein to β-actin.

### Immunohistochemistry assay

The slides of LUAD tissues were stained with rabbit anti-NEIL3 (1:400 dilution; cat. #PA5-51022; Thermo), followed by anti-rabbit secondary antibody (1:2000 dilution; cat. #ab205718; Abcam) and diaminobenzidine treatment. Immunohistochemistry score (IHC-score) = staining intensity score × staining area ratio score of positive cells. The staining intensity was scored as 0 for negative, 1 for weakly positive, 2 for medium positive, and 3 for strongly positive. The positive area ratio was scored as 0 (0%), 1 (1–25%), 2 (26–50%), 3 (51–75%), or 4 (76–100%) [24]. When the IHC-score was less than 6, the case was classified into the low expression group; otherwise, the case was classified into the high expression group. The staining results were independently scored by two pathologists.

### Statistical analysis

All statistical analyses were performed using R (v.3.5.3) and SPSS version 15.0 (SPSS Inc., Chicago, IL, USA). 22 pairs of tumor and normal tissues were used in Western blotting, which statistical power is 0.8291. 19 pairs of tumor and normal tissues were used in PCR, which statistical power is 0.8130. Statistical power calculations are performed using STATA v.15.0. To evaluate the correlation between NEIL3 expression and the other variables (sex, age, TNM stage, tumor size, distant metastasis, and OS), we performed Spearman correlation test, Chi-square Tests and Wilcoxon/Kruskal–Wallis test. We stratified the cohort into patients with a high or low median NEIL3 expression value or median risk score as the cut-off value. Multivariate Cox regression analysis was conducted to identify independent prognostic factors. Values of *p* < 0.05 were considered as statistically significant.

## Results

### NEIL3 overexpression in LUAD

**Fig. 1:**
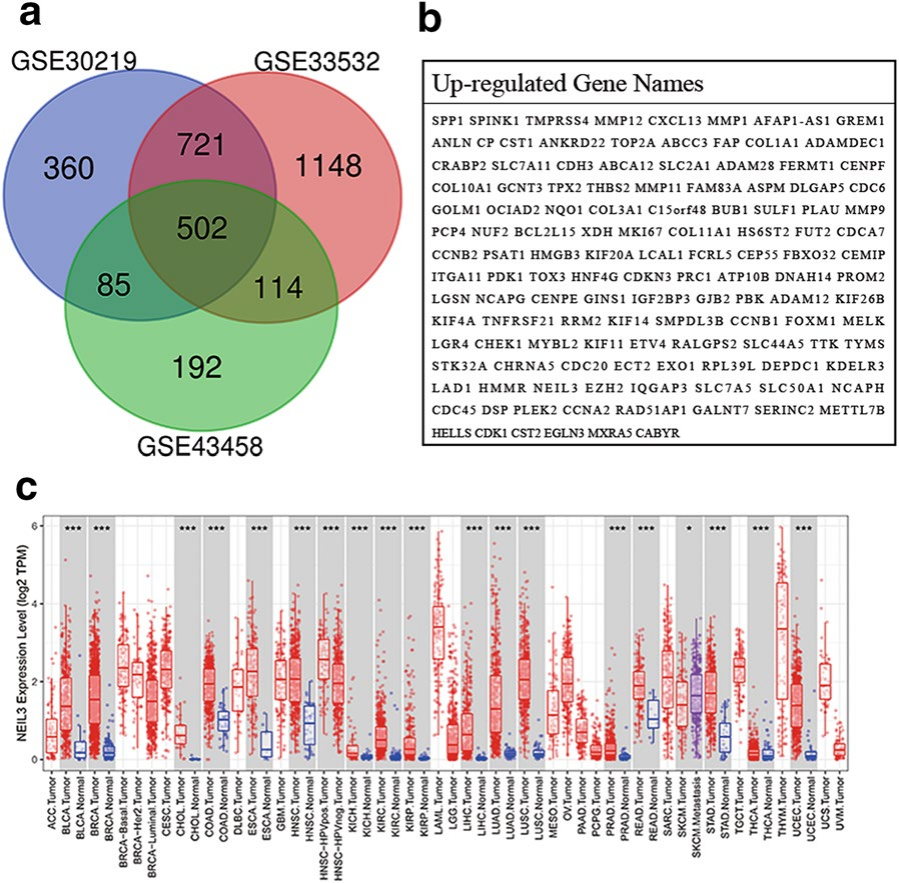
Identification of commonly upregulated genes. **a** Differentially expressed genes (DEGs) of GSE30219, GSE43458, and GSE33532 identified via GEO2R online tools and Venn diagram software. **b** The 126 overexpressed DEGs in lung adenocarcinoma. **c** NEIL3 expression in various cancer tissues and normal tissues. *p < 0.05, **p < 0.01, ***p < 0.001

We thoroughly screened the LUAD data in the GEO database and selected three miRNA-sequencing datasets: GSE30219, GSE43458, and GSE33532. We identified 502 common DEGs among them (Fig. 1a), including 126 up-regulated DEGs in cancer tissues (Fig. 1b). After an extensive literature review, we found that NEIL3 is a very promising DNA repair gene. TIMER data showed that NEIL3 expression increased in 19 kinds of tumor tissues compared to adjacent normal tissues, especially in LUAD (Fig. 1c**)**.

**Fig. 2:**
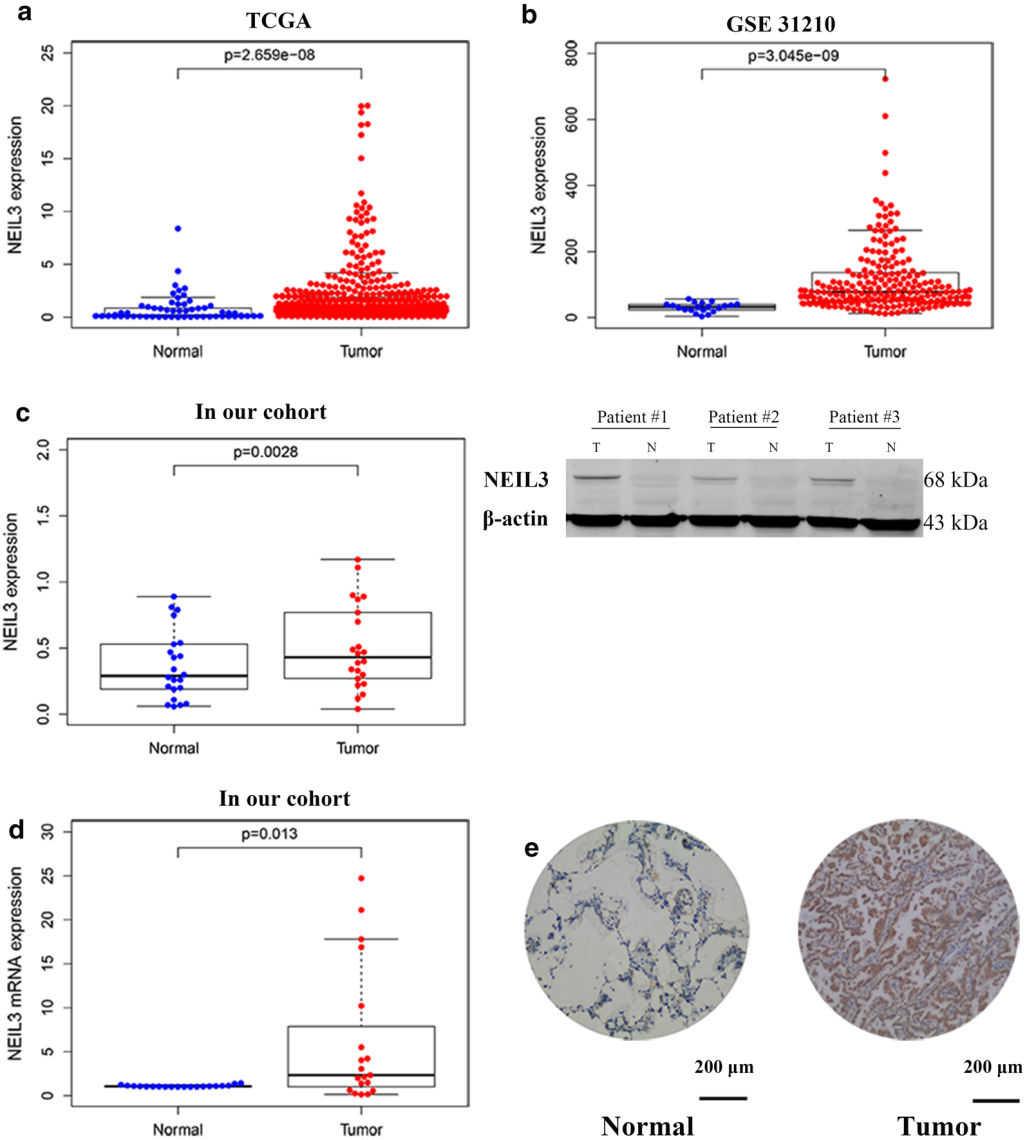
NEIL3 was overexpressed in LUAD. **a**, **b** The NEIL3 expression in TCGA LUAD cohort and GSE31210 cohort. **c** NEIL3 protein level in 22 pairs of LUAD tissues and their matched normal tissues were determined by western blotting. **d** NEIL3 mRNA levels were determined by quantitative real-time polymerase chain reaction (qRT-PCR). **e** Immunohistochemical staining for NEIL3 in normal lung tissue and lung adenocarcinoma tissues (original magnification ×100). *N* normal tissue, *T* tumor tissue

Analysis of the NEIL3 gene expression data in the TCGA and GSE31210 cohorts confirmed that NEIL3 is overexpressed in LUAD tissue (Fig. 2a, b). Therefore, RT-qPCR and western blotting were verified that the NEIL3 protein and mRNA levels were up-regulated in 22 LUAD tissues compared with the matched normal tissues (Fig. 2c, d). NEIL3 staining was positive in LUAD tissues but negative in normal lung tissue after immunohistochemistry staining (Fig. 2e). The results consistently showed that NEIL3 was obviously overexpressed in LUAD compared with the matched normal tissues (*p* < 0.001) (Fig. 2).

### Correlation between NEIL3 expression and clinicopathological characteristics of LUAD patients

**Fig. 3:**
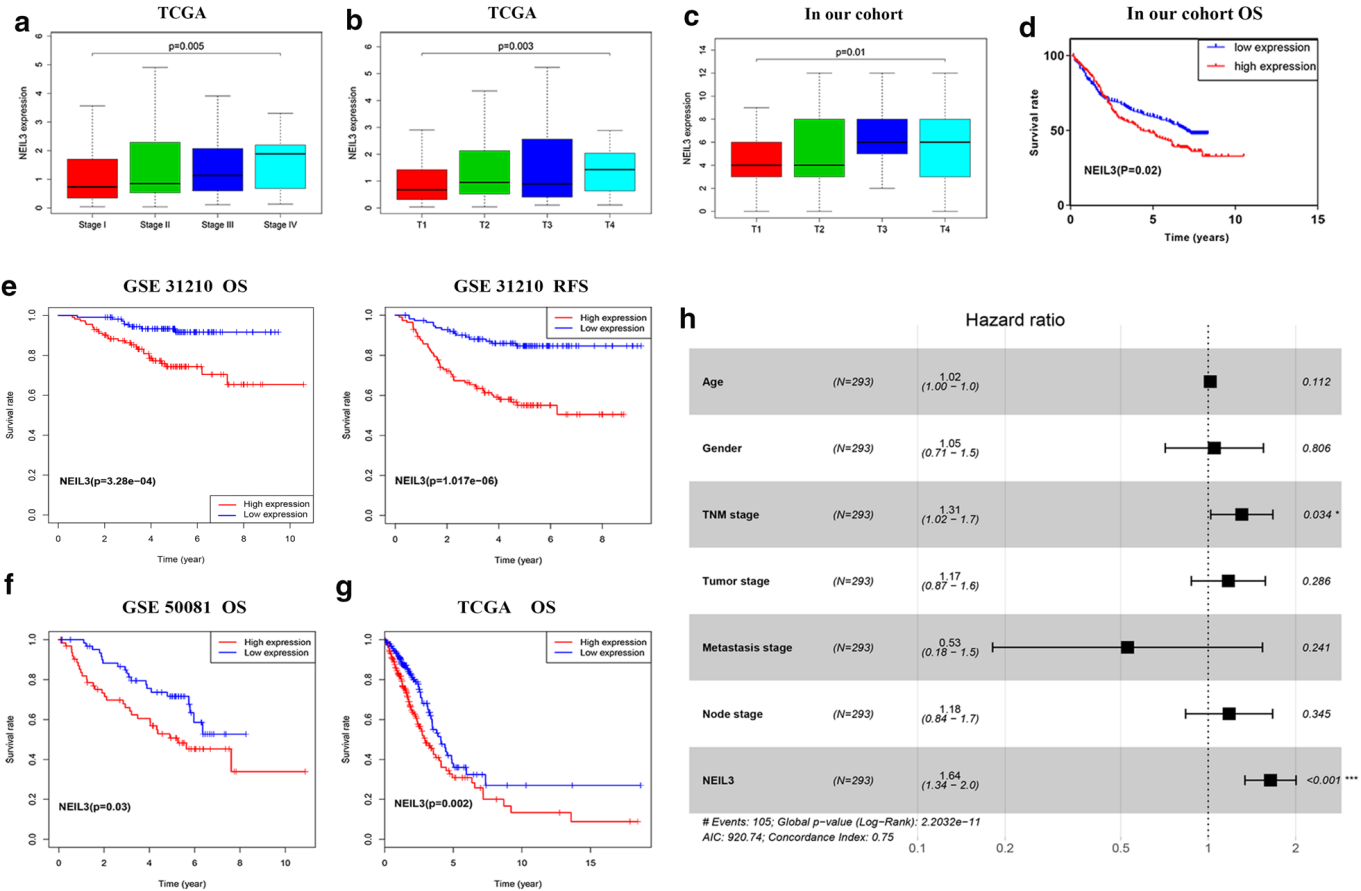
Association with NEL3 expression, clinicopathological characteristics, overall survival (OS), and relapse-free survival (RFS). **a**–**c** Increased NEIL3 expression was significantly associated with advanced TNM stage and larger tumor size. **d**–**g** The Kaplan–Meier plotters based on four datasets showed LUAD patients with high NEIL3 expression have poor OS and RFS. **h** The multivariate analyses proved the independent predictive ability of TNM stage and NEIL3 expression for LUAD overall survival

The characteristics of our 406 LUAD cohort subjects and 293 TCGA LUAD cohort subjects are summarized in Tables 1 and 2*.* All samples were divided into low and high expression groups by IHC-score and median expression level. The results revealed that NEIL3 overexpression was closely associated with advanced TNM stage (*p* < 0.05; Fig. 3a) and larger tumor size (*p* < 0.05; Fig. 3b, c). No significant correlation was noted between NEIL3 expression and other clinicopathological characteristics. Previous research has proved that NEIL3 could protect telomere integrity during the S phase and accurately segregate chromosomes in actively dividing cells [10], which may explain the advanced T classification in NEIL3-overexpressing patients.

**Table 1:** Relationship between NEIL3 expression and clinicopathology in the TCGA LUAD cohort

Characteristics	n	NEIL3		χ^2^	P value
		Low expression	High expression		
Total	293	147	146		
Age				0.206	0.65
≤ 60	98	51 (52.05)	47 (47.95)		
> 60	195	96 (49.24)	99 (50.76)		
Gender				2.13	0.144
Male	138	63 (45.65)	75 (54.35)		
Female	155	84 (54.19)	71 (45.81)		
TNM stage				7.689	0.004*
I and II	223	122 (54.70)	101 (45.30)		
III and IV	70	25 (35.70)	45 (64.30)		
Tumor stage				7.201	0.027*
I	88	54 (61.40)	34 (38.60)		
II	169	80 (47.30)	89 (52.70)		
III and IV	37	14 (37.80)	23 (62.20)		
Metastasis stage				2.809	0.094
Negative	274	141 (51.50)	133 (48.50)		
Positive	19	6 (31.60)	13 (68.40)		
Node stage				2.259	0.133
Negative	187	100 (53.50)	87 (46.50)		
Positive	106	47 (44.30)	59 (55.70)		

*Statistically significant

**Table 2:** Relationship between NEIL3 expression and clinicopathology in our LUAD cohort

Characteristics	n	NEIL3		χ^2^	P value
		Low expression	High expression		
Total	406	228	178		
Age				1.082	0.298
≤ 60	146	77 (52.74)	69 (47.26)		
> 60	260	151 (58.08)	109 (41.92)		
Gender				2.814	0.093
Male	249	148 (59.44)	101 (40.56)		
Female	157	80 (50.96)	77 (49.04)		
TNM stage				1.81	0.178
I and II	312	169 (54.17)	143 (45.83)		
III and IV	94	59 (62.77)	35 (37.23)		
Tumor stage				14.648	0.002*
I	203	124 (61.08)	79 (38.92)		
II	145	81 (55.86)	64 (44.14)		
III	32	8 (25.00)	24 (75.00)		
IV	26	15 (57.69)	11 (42.31)		
Metastasis stage				0.001	0.971
Negative	397	223 (56.17)	174 (43.83)		
Positive	9	5 (55.56)	4 (44.44)		
Node stage				0.88	0.348
Negative	222	120 (54.05)	102 (45.94)		
Positive	184	108 (58.70)	76 (41.30)		

*Statistically significant

### NEIL3 overexpression predicts poor prognosis in LUAD patients

This study included all follow-up survival information of the GSE50081, GSE31210, TCGA datasets and our cohort. As is shown in Fig. 3d–g, Kaplan–Meier curve and log-rank test analyses indicated that up-regulated NEIL3 expression significantly reduced the OS and RFS of LUAD patients (*p* < 0.05). All risk factors and NEIL3 expression levels were subjected in the univariate Cox regression analysis (Table 3); of them, these were associated with OS: TNM stage, tumor size, lymph node metastasis, and NEIL3 expression (*p* < 0.001). Moreover, the multivariate Cox analysis result showed that increased NEIL3 expression [hazard ratio (HR) = 1.638, 95% confidence interval (CI) 1.338–2.005; *p* < 0.001] and advanced TNM stage (HR = 1.305, 95% CI 1.021–1.669; *p* < 0.05) were independent predictors of poor prognosis (Table 3 and Fig. 3h).

**Table 3:** Univariate and multivariate analyses of factors associated with overall survival in LUADs using Cox regression

Variable	Univariate		Multivariate	
	HR (95% CI)	P	HR (95% CI)	P
Age	1.004 (0.985 to 1.023)	0.707	1.016 (0.996 to 1.037)	0.112
Gender	1.105 (0.753 to 1.621)	0.611	1.050 (0.712 to 1.549)	0.806
TNM stage	1.322 (1.202 to 1.454)	0.000*	1.305 (1.021 to 1.669)	0.034*
Tumor stage	1.696 (1.348 to 2.134)	0.000*	1.173 (0.875 to 1.573)	0.286
Metastasis stage	1.594 (0.871 to 2.918)	0.13	0.528 (0.181 to 1.537)	0.241
Node stage	1.820 (1.474 to 2.247)	0.000*	1.180 (0.836 to 1.666)	0.345
NEIL3	1.760 (1.456 to 2.129)	0.000*	1.638 (1.338 to 2.005)	0.000*

*Statistically significant

### NEIL3 expression is associated with immune cell infiltration in LUAD

**Fig. 4:**
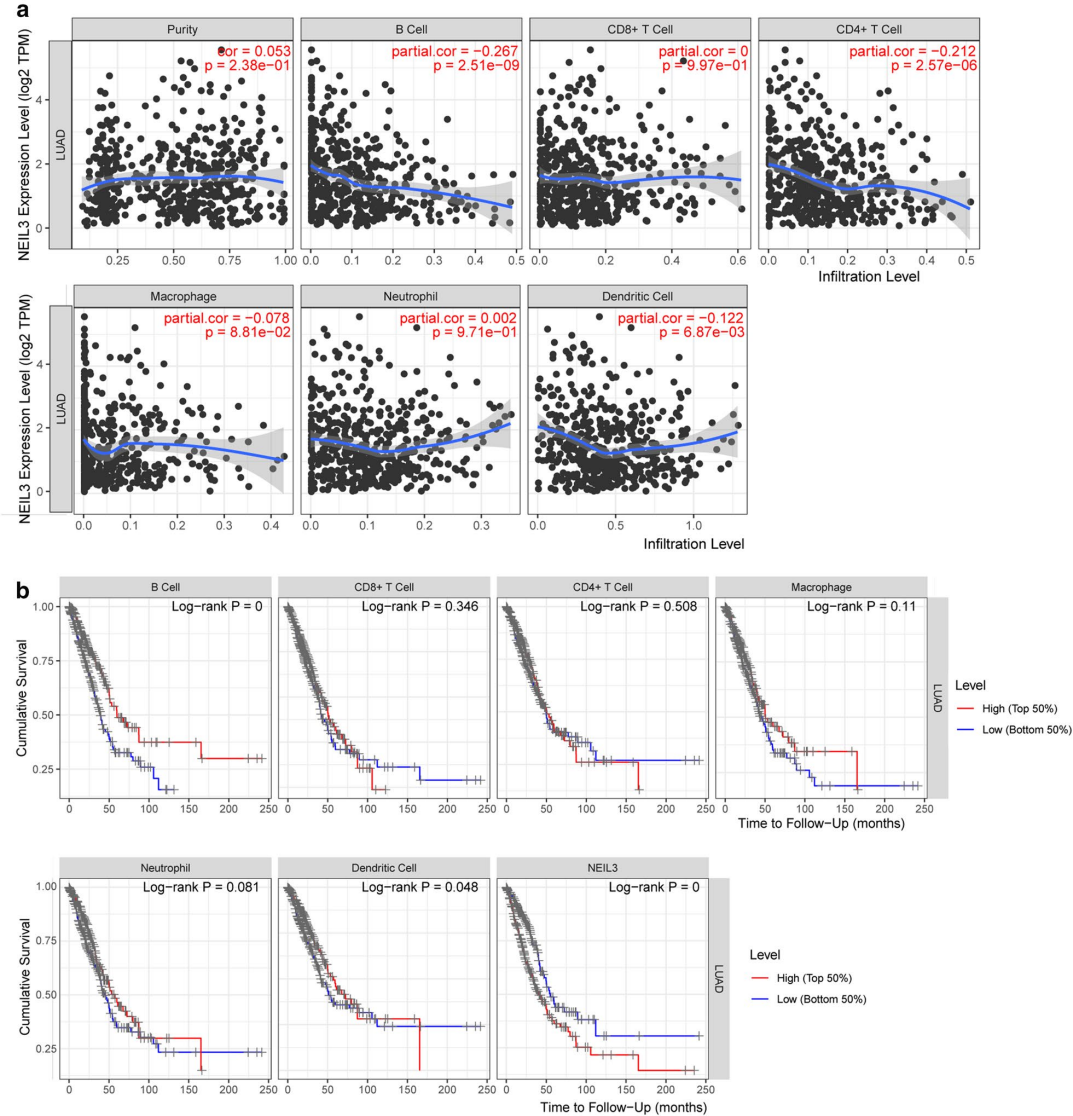

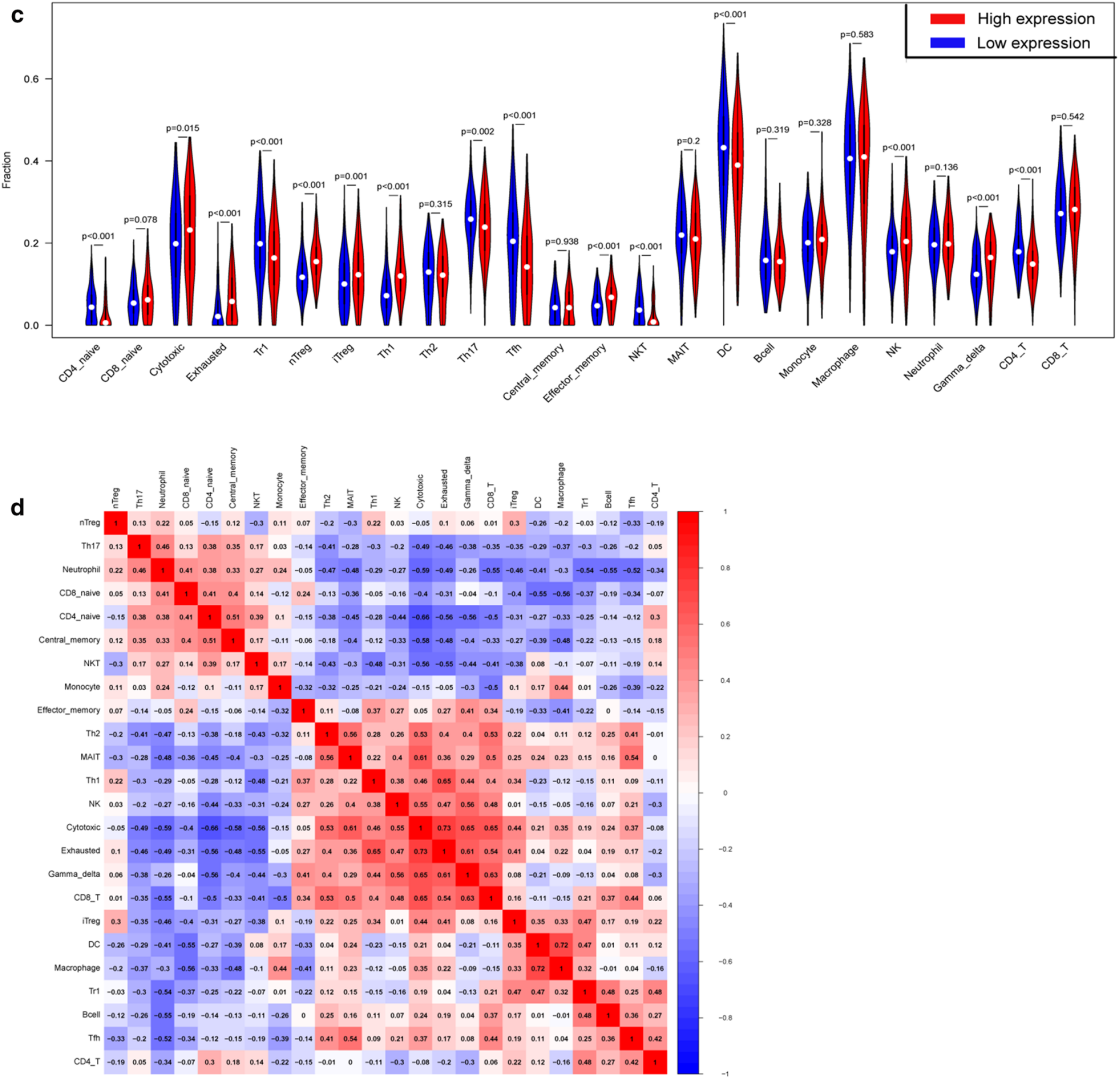
Association with NEIL3 expression and tumor-infiltrating immune cells (TIICs). **a** Correlations between NEIL3 expression and immune infiltration levels from TIMER web tool. **b** Association between clinical outcome and abundance of immune infiltrates or NEIL3 expression in the TIMER “Survival” module. **c** Proportions of 24 tumor-infiltrating immune cells in high and low NEIL3 expression groups. **d** Correlation matrix of all 24 immune cell proportions

Patients with the same histological type of cancer may have different degree of immune infiltration cells that lead to diverse clinical outcomes [22, 25]. The fact that an increased number of tumor-infiltrating lymphocytes in primary tumor tissue relates to good prognosis has been reported in several cancers, including LUAD [26]. The TIMER result showed that NEIL3 expression had a significant negative correlation with the infiltration of B cells, CD4+ T cells, and DCs (*p* < 0.05; Fig. 4a). The TIMER “Survival” module showed that high infiltrating levels of B cells benefit OS in contrast to NEIL3 expression (*p* < 0.05; Fig. 4b). We speculated that NEIL3 overexpression could affect OS by regulating the degree of B-cell infiltration in LUAD.

The ImmuCellAI analysis showed that the frequencies of natural T regulatory (nTreg), induced T regulatory (iTreg), Th1 cells, exhausted T cells, NK cells, effector memory and Gamma delta T cells exhibited a positive correlation with NEIL3 expression (*p* < 0.001), whereas the proportion of CD4 naïve cells, Tregulatory1 (Tr1), Th17, follicular helper T cell (Tfh), NK T, DC, and CD4 T cells exhibited a negative correlation (*p* < 0.001) (Fig. 4c). This funding suggested that NEIL3 has a regulatory effect on the formation of the LUAD immune microenvironment, especially on T-cell subsets, NK cells, and DCs. Figure 4d shows the correlation between different types of immune cell subsets. Exhausted T cells had the strongest positive correlation with Cytotoxic T lymphocyte (Pearson correlation = 0.73), while Cytotoxic T lymphocyte had the strongest negative correlation with CD4 naïve cells (Pearson correlation = − 0.66). Taken together, these findings indicate that NEIL3 plays a key role in the regulation of immune-infiltrating cells in LUAD.

### KEGG and GO enrichment analysis of NEIL3 and co-expressed genes in LUAD

**Fig. 5:**
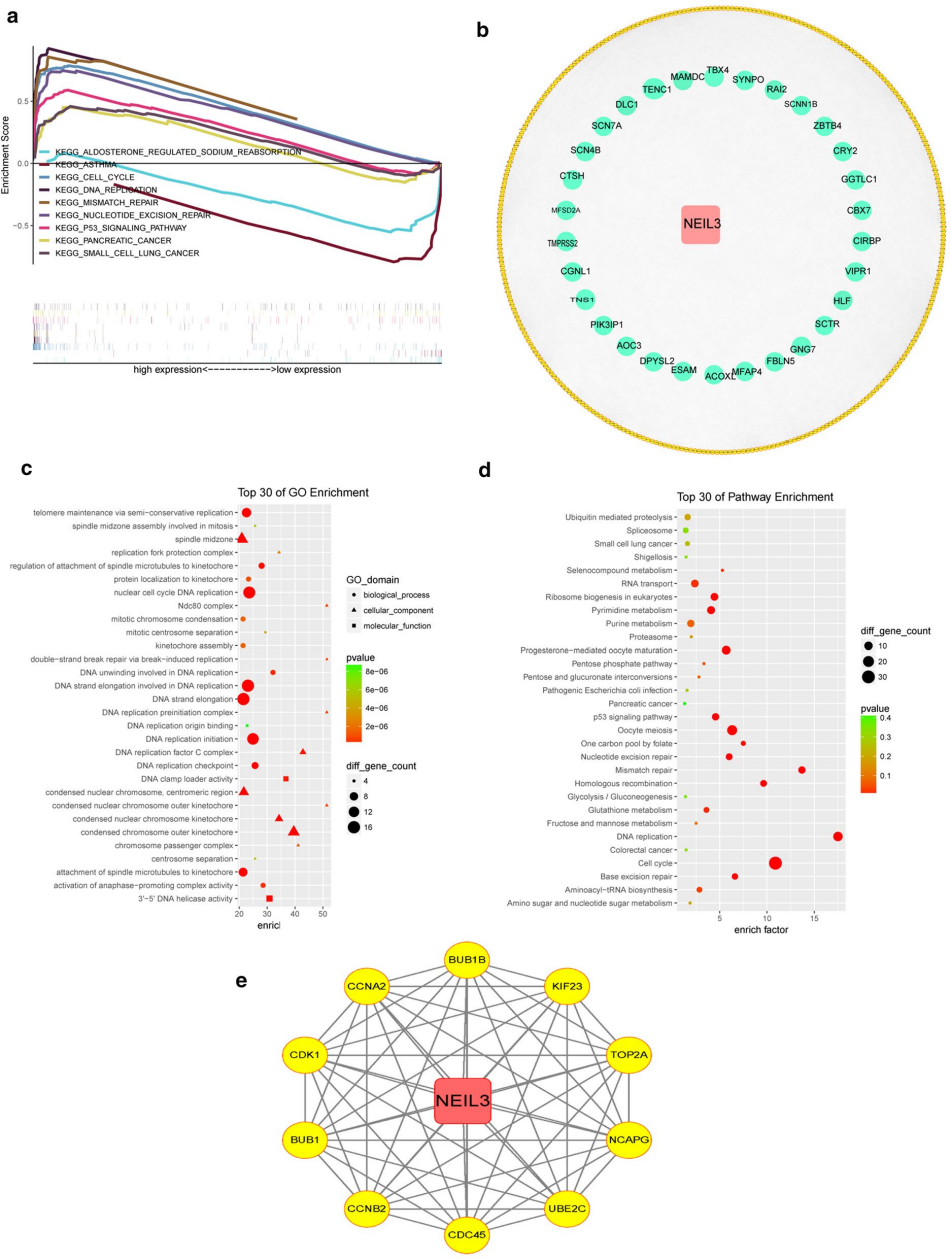
Functional enrichment analysis and protein–protein interaction network of NEIL3 and genes co-expressed with NEIL3. **a** KEGG signaling pathway enrichment analysis of the high and low NEIL3 expression groups via GSEA software. **b** The PPI network among genes co-expressed with NEIL3. The yellow dots indicate a positive correlation with NEIL3, whereas the blue dots indicate a negative correlation. **c**, **d** KEGG enrichment analysis and GO analysis of the co-expressed genes. **e** The 10 hub genes among the 370 co-expressed genes identified by the Cytoscape software CytoHubba plugin

According to FDR < 0.050 and normalized enrichment score, the GSEA analysis revealed that the pathways of the cell cycle, nucleotide excision repair, DNA replication, mismatch repair, and the P53 signaling pathway were enriched in the high NEIL3 expression phenotype (Fig. 5a). The asthma and aldosterone-regulated sodium reabsorption pathways were enriched in the low NEIL3 expression cohort (Fig. 5a). It is worth noting that the cell cycle pathway, playing a crucial part in tumorigenesis and development, is associated with NEIL3 expression.

Subsequently, we analyzed mRNA sequencing data of TCGA cohort and acquired 370 genes co-expressed with NEIL3 (Additional file 3: Table S1, absolute Pearson correlation coefficient > 0.5, *p* < 0.001). As showed in the protein–protein interaction network (PPI), there were 323 genes (yellow dots) positively related to NEIL3, versus 47 genes (blue dots) negatively related to NEIL3 (Fig. 5b). Next, we performed GO and KEGG enrichment analyses and displayed the top 30 items. The GO analysis indicated that the co-expressed genes were significantly enriched in nuclear cell cycle DNA replication, DNA strand elongation, and DNA replication initiation in the biological process group; condensed chromosome outer kinetochore and spindle midzone in the cellular component group; 3′–5′ DNA helicase activity in the molecular function group (Fig. 5c). Similarly, the KEGG pathway enrichment analyses showed that these genes were mainly enriched in the cell cycle, oocyte meiosis and DNA replication (Fig. 5d). Taking 370 co-expressed genes into analysis via the Cytoscape software cytoHubba plugin, we filtered 10 hub genes according to node degree: TOP2A, CCNA2, BUB1B, CDC45, BUB1, CDK1, NCAPG, KIF23, UBE2C, and CCNB2 (Fig. 5e, Table 4). Several hub genes (CDK1, CCNA2, CDC45, and CCNB2) played a vital role in cell cycle progression, whereas genomic instability contributed to potentiate tumorigenesis [27].

**Table 4:** The hub genes co-expressed with NEIL3

Gene	Cor	P value	Group
TOP2A	0.635	1.52E−63	Positive
CCNA2	0.726	2.36E−91	Positive
BUB1B	0.683	4.49E−77	Positive
CDC45	0.631	2.04E−62	Positive
BUB1	0.669	7.54E−73	Positive
CDK1	0.668	1.34E−72	Positive
NCAPG	0.7	2.21E−82	Positive
KIF23	0.672	1.49E−73	Positive
UBE2C	0.591	3.82E−53	Positive
CCNB2	0.674	3.35E−74	Positive

### Establishment and evaluation of prognostic signature for LUAD patients

Based on the TCGA LUAD cohorts, we verified that all 10 of hub genes were conspicuously overexpressed in LUAD tissues versus normal lung tissues (*p* < 0.05; Additional file 1: Fig. S1). In addition, the high expressions of nine hub genes (all but TOP2A) were remarkably associated with poor prognosis in LUAD patients (p < 0.05; Additional file 2: Fig. S2).

**Fig. 6:**
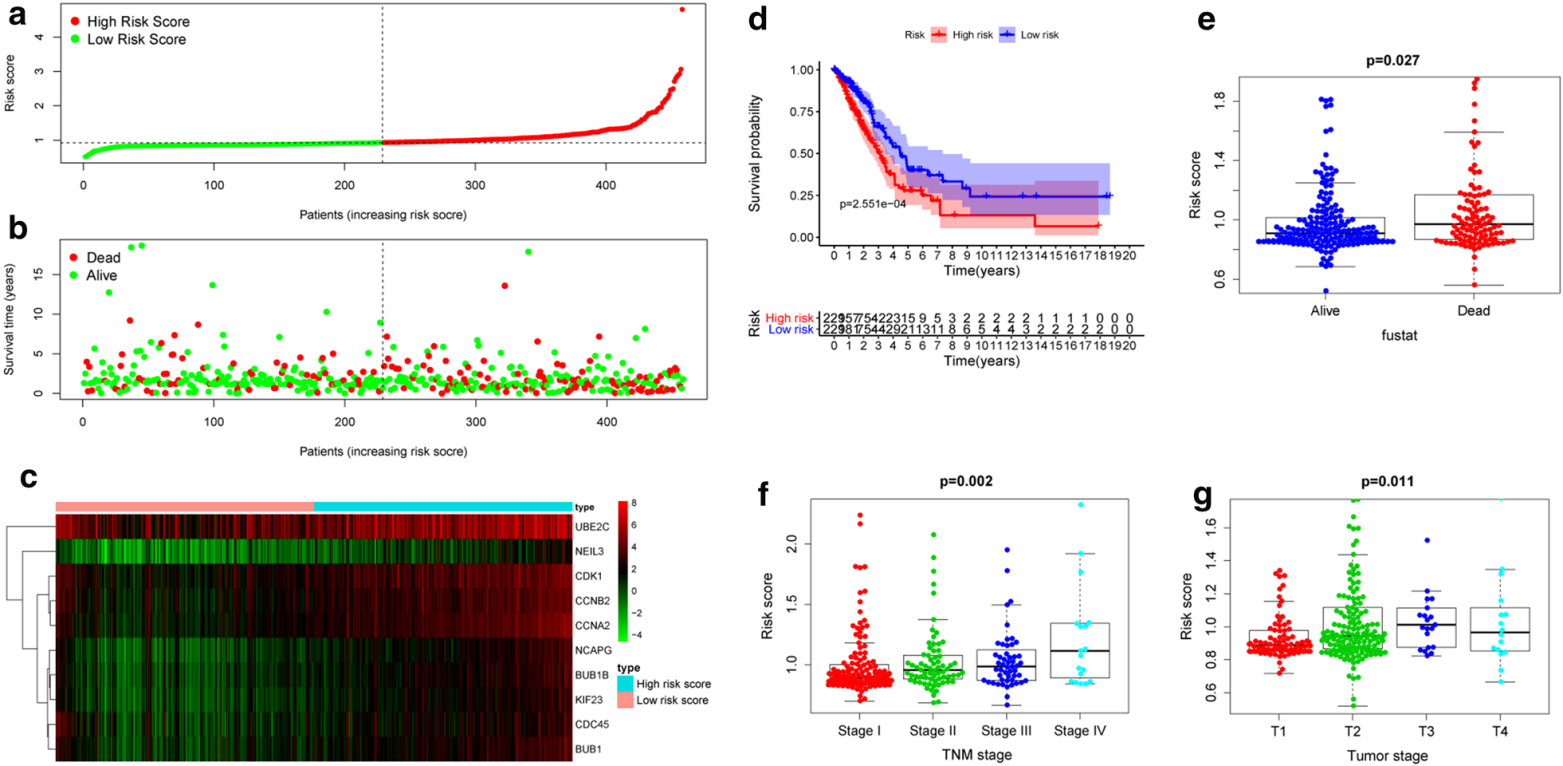
Relation between signature and cancer risk. **a** Distribution of risk score. **b** Proportion of deaths was increased in the high risk score group. **c** Hierarchical clustering of the 10 genes between the low and high risk groups. Red, up-regulated; green, down-regulated. **d**–**g** A higher risk score was remarkably associated with shorter overall survival, poorer clinical outcomes, more advanced TNM stage, and larger tumor size (*p* < 0.05)

Next, we attempted to establish a prognostic signature based on the expressions of NEIL3 and the other nine hub genes (Table 4). According to the median risk score (*cut-off* = 0.926), the LUAD patients were divided into two groups with discrete clinical outcomes for OS. Figure 6a shows the distribution of risk scores in the LUAD dataset. More deaths occurred in the high risk-score group than in the low risk score group (Fig. 6b). Meanwhile, the expressions of these 10 genes were up-regulated in the high-risk group (Fig. 6c). Figure 6d shows that patients in the low-risk group had significantly better OS than others in the Kaplan–Meier analysis (*p* < 0.05). Analysis of the association between risk core and various clinical features revealed that an increased risk score was correlated with more advanced stage, the larger tumor size and poor outcome (Fig. 6e, f, g).

**Fig. 7:**
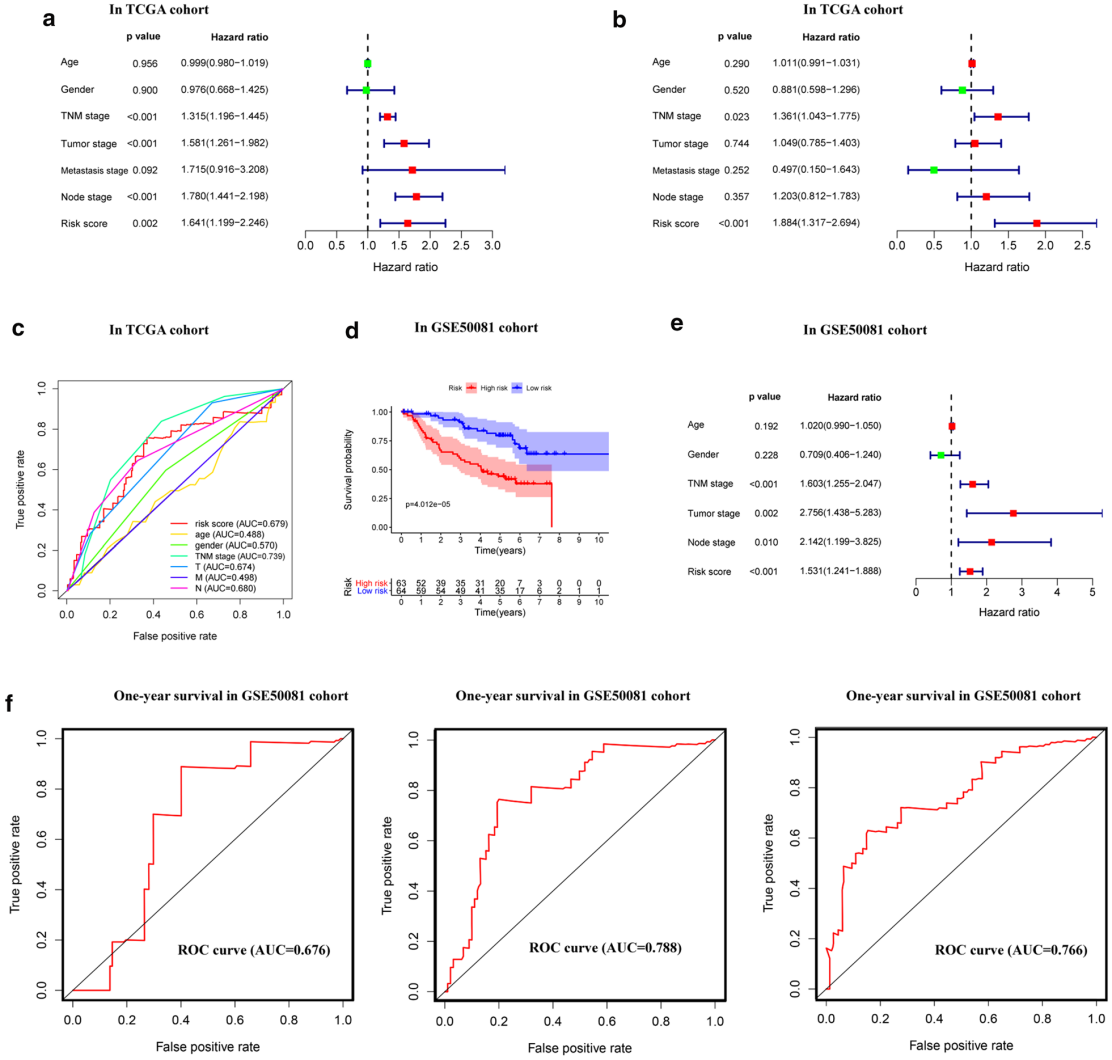
Predictive performances and verification of signature. A forest plot of univariate (**a**) and multivariate (**b**) Cox regression analyses revealed that risk score could be an independent prognostic factor in TCGA cohort. **c** The area under the curve (AUC) of the multiple receiver operating characteristic (ROC) curves for multiple valuable factors in TCGA cohort. **d** The Kaplan–Meier plotters of the test cohort (GSE50081 dataset) showed LUAD patients in the high risk score group have poor overall survival. **e** Multivariate Cox regression analysis of the GSE50081 cohort. **f** 1-, 3-, and 5-year ROC curves of the GSE50081 cohort

The univariate and multivariate Cox analyses revealed that the risk score of a 10-gene signature was an independent prognostic factor for LUAD patients (*p* < 0.05, Fig. 7a, b). After ROC analysis, we observed a marked predictive advantage of the risk-score signature (area under the curve [AUC] = 0.679), stage (AUC = 0.739) and lymph node metastasis (AUC = 0.680) (Fig. 7c).

**Table 5:** Univariate and multivariate analyses of factors in the GSE50081 cohort using Cox regression

Variable	Univariate		Multivariate	
	HR (95% CI)	P	HR (95% CI)	P
Age	1.020 (0.990 to 1.050)	0.192	1.009 (0.978 to 1.041)	0.562
Gender	0.709 (0.406 to 1.240)	0.228	0.701 (0.395 to 1.244)	0.225
TNM stage	1.603 (1.255 to 2.047)	0.000*	0.904 (0.126 to 6.476)	0.92
Tumor stage	2.756 (1.438 to 5.283)	0.002*	2.229 (0.204 to 24.396)	0.512
Node stage	2.142 (1.199 to 3.825)	0.010*	2.940 (0.056 to 155.468)	0.594
Risk score	1.531 (1.241 to 1.888)	0.000*	1.577 (1.203 to 2.067)	0.001*

*Statistically significant

Finally, we selected a test cohort based on GSE50081 (127 LUAD patients with TNM stage I & II). In line with the TCGA cohort, the risk score of the 10-gene prognostic signature could be an independent prognostic factor for LUAD patients (*p* < 0.001; Fig. 7e, Table 5). Patients in the high risk score group had poor OS (*p* < 0.001; Fig. 6d), and the AUC values of the 1-, 3-, and 5-year ROC curves were 0.676, 0.788, and 0.766, respectively (*p* < 0.001; Fig. 7f). These evidences strongly suggests that the 10-gene prognostic signature has superior diagnostic accuracy and may benefit LUAD patients in the early stage.

## Discussion

LUAD, the most common type of malignant tumors, has significant morbidity and mortality rates [28]. Growing scientific research has focused on searching for effective treatment methods and sensitive biomarkers to improve the 5-year survival rate and life quality of LUAD patients. NEIL3, a DNA glycosylase of the BER pathway, repairs telomere oxidative damage and protects telomere integrity in cells with a high proliferative capacity during the S phase, which may explain the advanced Tumor stage in NEIL3 overexpressing patients [17]. In addition, some studies indicated NEIL3 as a cell cycle dependent gene was regulated by BRG1 in breast cancer cells and that NEIL3 overexpression may facilitate distant metastasis in primary melanoma [15, 19]. Tran et al. found NEIL3 was overexpressed in a variety of tumors such as pancreatic adenocarcinoma, lower grade glioma, and kidney papillary cell carcinoma; furthermore, NEIL3 overexpressed tumors accumulate mutation and chromosomal variations [29]. As mentioned in the literature review, NEIL3 plays a crucial role in preventing autoimmunity and cell proliferation [11, 13]. Here a comprehensive bioinformatics analysis was performed based on gene transcript profiles of LUAD from TCGA and GEO databases. Combined with the IHC-scores of our 406 patients cohort, NEIL3 expression was up-regulated in LUAD tissues and correlated with clinicopathological characteristics, especially advanced TNM stage and large tumor size. Meanwhile, the Cox regression analysis results demonstrated that NEIL3 may serve as an independent prognostic predictor in LUAD patients.

Over the past decade, immunotherapy has been a well-known promising cancer treatment with amazing achievements in treating various refractory malignancies. The pivotal strategy of immunotherapy is to interfere with immune checkpoints expressed in immune cells [30]. Immune cells are a crucial part of the immune microenvironment, including tumor infiltrating lymphocytes (TILs), tumor-associated macrophages (TAM), dendritic cells (DC), and myeloid-derived suppressor cells (MDSCs) [31, 32]. As reported in the immunoediting theory, tumor invasive immune cells (TIICs) play a “double-edged sword” role in the development of cancers. A large number of studies have found the occurrence and development of lung adenocarcinoma not only depends on the lung cancer cells themselves but also is regulated by the tumor-infiltrating immune cells in the lung cancer microenvironment [33].

Based on the TIMER database review, NEIL3 expression had a significant negative correlation with B cells, CD4^+^ T cells, and DCs. The ImmuCellAI analysis revealed nTreg, iTreg, and Exhausted T cells were increased in the high NEIL3 expression group, whereas Th17 cells, DCs and CD4^+^ T cells were decreased. Th17 cells play a contradictory role in tumorigenesis and might be associated with secreting cytokines such as IL-17A, IL-17F, IL-21, and IFN-γ + Th17 cells as well as effector lymphocytes, including Th1, Tc1, and NK cells [34–36]. Treg cells could inhibit the activation of T lymphocytes by secreting cytokines such as IL-4, IL-10, and TGF-β, which regulate the immune function of tumor patients and promote the proliferation of lung cancer cells [37]. Evidence indicates that DCs induced antitumor immunity and inhibit the formation of new blood vessels in tumors in the lung cancer microenvironment [38]. These findings suggest that NEIL3 overexpression could increase the proportion of T regulatory cells and inhibit the antitumor function of Th17 cells and DCs, which predicted a poor OS and advanced TNM stage. Together this evidence demonstrates that NEIL3 plays a pivotal role in the regulation of immune infiltrating cells and could be considered a novel immune-related therapeutic target in LUAD. The molecular mechanism underlying NEIL3 gene and tumor microenvironment is still not known, which is our limitation and next crucial research question.

Equally important, GSEA analysis showed that the cell cycle and P53 signaling pathway were enriched in cases of high NEIL3 expression. As we all know, cell cycle proteins dysregulations is related to uncontrolled proliferation of a malignant tumor and becomes an attractive target in cancer therapy [39]. This work selected nine prognosis-associated hub genes among 370 genes co-expressed with NEIL3: CCNA2, BUB1B, CDC45, BUB1, CDK1, NCAPG, KIF23, UBE2C and CCNB2 (Table 3, Additional file 2: Fig. S2). CDK1, a cyclin-dependent kinase, plays an important part in regulating cell cycle progression and reportedly increases cellular proliferation in various cancers if dysregulated [40]. CCNA2 and CCNB2 are cyclin family proteins. CCNA2 was recognized as an effective prognostic marker in prostate, colon, lung, and liver cancers [41, 42]. In line with CCNA2, CCNB2 increased the risk of multiple cancer prognoses such as such as adrenocortical carcinoma [43], lung cancer [44], breast cancer [45], and colorectal adenocarcinoma [46].

Our result showed that NEIL3 and nine hub genes were in a tight co-expressed relationship. Combining the expression levels of the nine hub genes and NEIL3, a 10-gene prognostic signature was constructed to accurately predict the prognostic risk of LUAD patients. Multivariate Cox regression analyses proved that the risk score was an independent prognostic factor correlated with TNM stage and tumor size. Analysis of the test cohort data achieved the same results. As early as 2007, Chen et al. attempted to develop gene signatures correlated with NSCLC clinical outcomes and finally developed a five-gene prognostic signature for NSCLC [47]. Since then, scholars have performed a great number of studies to explore novel gene signature as effective classifier to predict prognosis. Sun et al. [48] identified a immune-related prognostic signature, but the AUC of verification set GSE50081 was only about 0.617, and Wang et al. [49] identified a 4-gene signature, but the average AUC was less than 0.7. In addition, Liu et al. [50] identified a 22-gene signature through analysis of autophagy gene expression. Although the AUC is high, the large number of genes that needs to be detected makes this analysis impractical for clinical use. By contrast, our signature has a high AUC using 10 genes, which makes it conducive to clinical application and provides a theoretical basis for the development of new targeted therapies.

NEIL3 expression and the 10-gene signature were proven to be independent predictors for LUAD patients using bioinformatics technology in this study. However, identifying NEIL3 in LUAD cell lines, exploring the underlying tumorigenesis mechanism, and designing novel potent selective NEIL3-targeted drugs require further research.

## Conclusion

In summary, this study revealed that NEIL3 was overexpressed in LUAD and identified it as a new diagnostic biomarker for LUAD patients. Increased NEIL3 expression was related to advanced stage and larger tumor size as an independent diagnostic factor of poor prognosis in LUAD patients. Moreover, NEIL3 may play an important role in regulating immune-infiltrating cells such as regulatory T cells and DCs and may affect LUAD cell proliferations. The cell cycle and P53 signaling pathway is the major pathway affected by NEIL3 in LUAD. And finally, we constructed a 10-gene prognostic signature (NEIL3 and nine co-expressed hub genes) to accurately predict prognostic risk of LUAD patients. The results based on the test cohort proved that the 10-gene signature had precise diagnostic accuracy. Meanwhile, NEIL3 could be a promising biomarker for diagnosis and treatment and correlates with immune infiltration in LUAD.

## Supplementary Information

**Additional file 1: Figure S1.** The expression levels of the 10 hub genes in LUAD tissues and normal lung tissues.

**Additional file 2: Figure S2.** The overall survival Kaplan–Meier plotters of the 10 hub genes in TCGA LUAD cohort.

**Additional file 3: Table S1.** The 370 significant co-expressed genes.

## Data Availability

The datasets of this study can be found in the Gene Expression Omnibus (GEO) repository (https://www.ncbi.nlm.nih.gov/gds/) and The Cancer Genome Atlas (https://www.ncbi.nlm.nih.gov/gds/).

## Abbreviations

LUAD: Lung adenocarcinoma
NEIL3: DNA endonuclease VIII-like 3
GEO: Gene expression omnibus
TCGA: The cancer genome atlas
RT-qPCR: Real-time quantitative PCR
GO: Gene ontology
KEGG: Kyoto encyclopedia of genes and genomes
PPI: Protein–protein interaction
OS: Overall survival
RFS: Relapse-free survival
IHC-score: Immunohistochemistry score
TIICs: Tumor-infiltrating immune cells
ImmuCellAI: Immune Cell Abundance Identifier
TIMER: Tumor Immune Estimation Resource
nTreg: T regulatory cells
iTreg: Induced T regulatory cells
AUC: The area under the curve
CI: Confidence interval
