# Supplementary figures and images for: NEIL3 may act as a potential prognostic biomarker for lung adenocarcinoma
Source: Cancer Cell Int. 2021 Apr 20;21:228. doi: 10.1186/s12935-021-01938-4 (PMC8059184; doi:10.1186/s12935-021-01938-4)

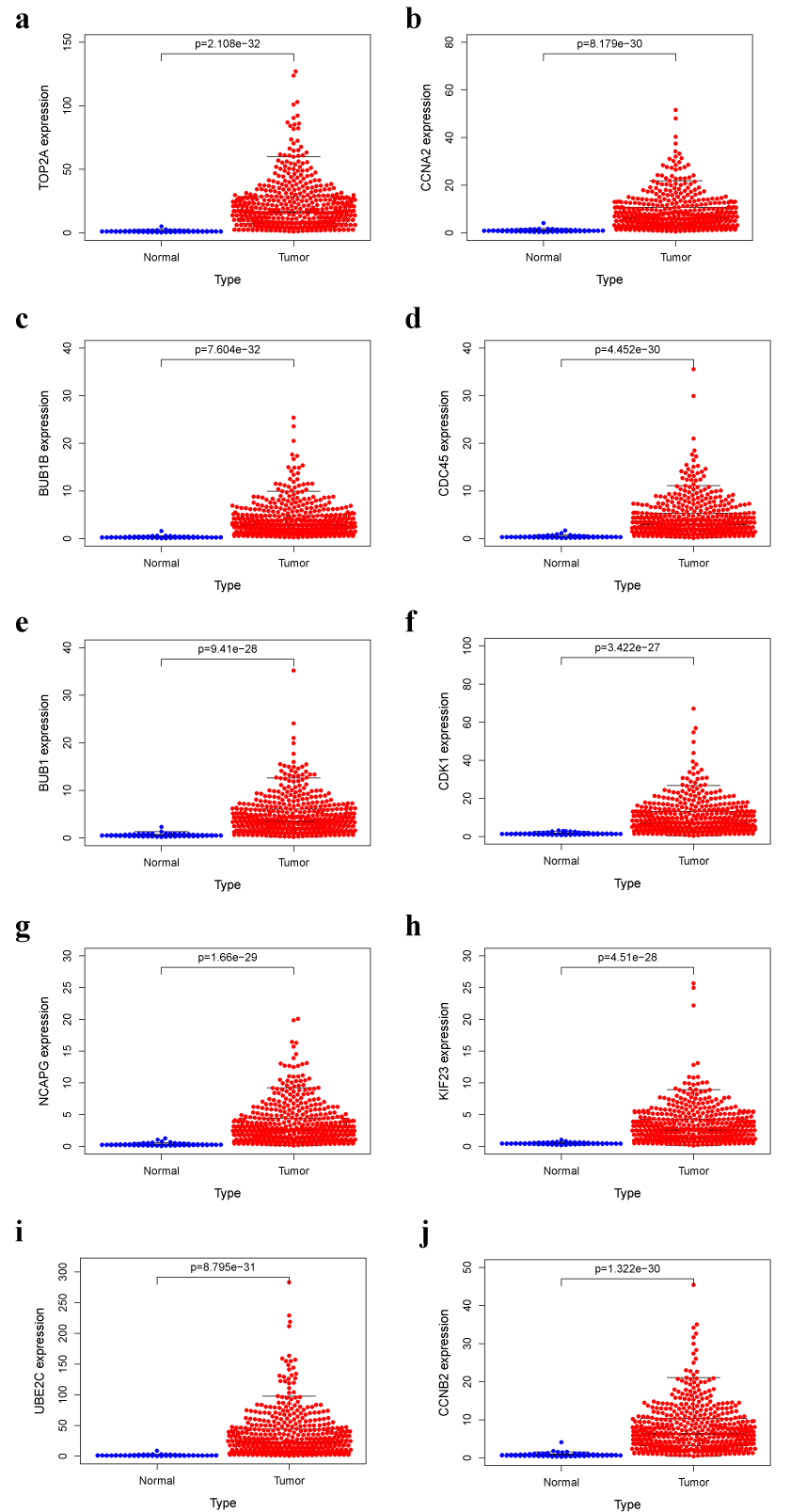

Supplement: Supplementary file 1 — Additional file 1: Figure S1. The expression levels of the 10 hub genes in LUAD tissues and normal lung tissues. [file 12935_2021_1938_MOESM1_ESM.tif]

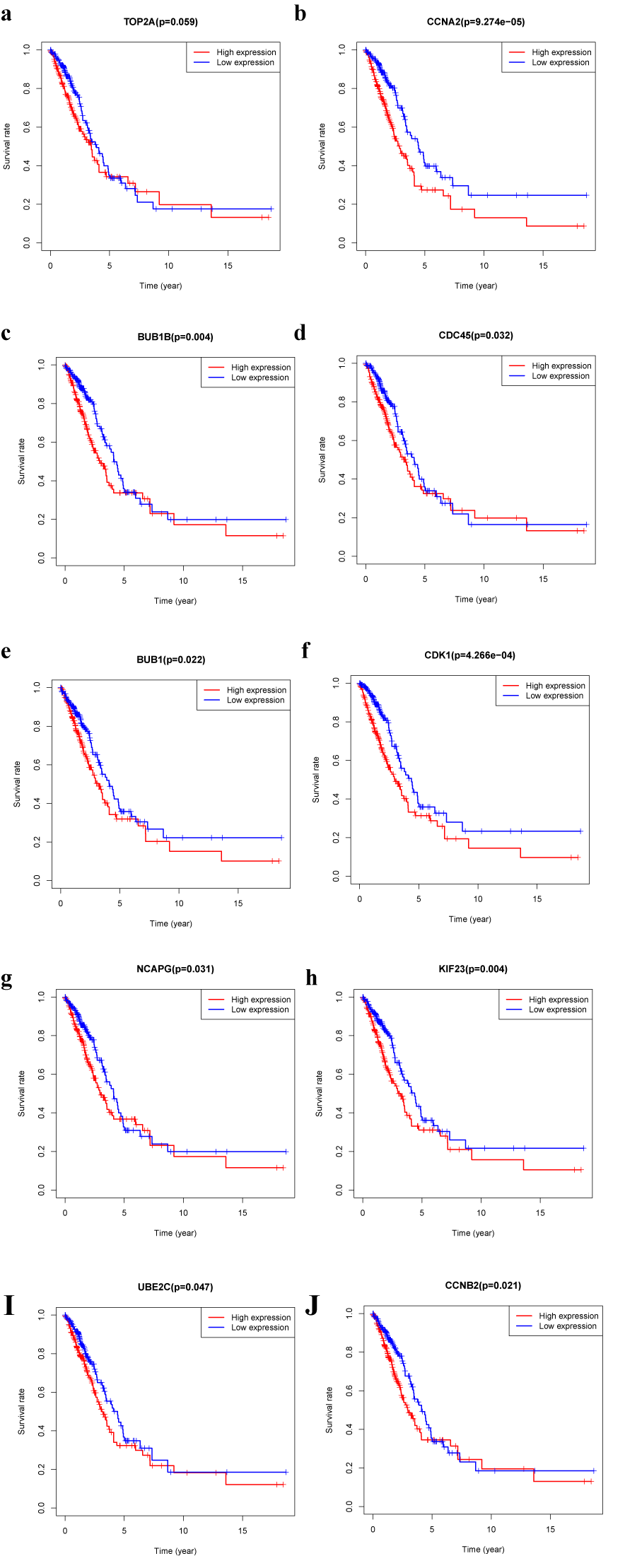

Supplement: Supplementary file 2 — Additional file 2: Figure S2. The overall survival Kaplan–Meier plotters of the 10 hub genes in TCGA LUAD cohort. [file 12935_2021_1938_MOESM2_ESM.tif]
